# Isolation and Identification of Fisetin: An Antioxidative Compound Obtained from *Rhus verniciflua* Seeds

**DOI:** 10.3390/molecules27144510

**Published:** 2022-07-14

**Authors:** Su-Hwan Kim, Chang-Ki Huh

**Affiliations:** 1 Research Institute of Food Industry, Sunchon National University, Suncheon 57922, Korea; suhwan010@sunchon.ac.kr; 2 Department of Food Science and Technology, Sunchon National University, Suncheon 57922, Korea

**Keywords:** *Rhus verniciflua*, antioxidative activities, antioxidative compound, fisetin

## Abstract

The goal of this study was to provide basic data for the development of functional food and health materials for *Rhus verniciflua* (*R. verniciflua*) seeds. We investigated an antioxidative compound obtained from these seeds. Solvent fractionation was carried out on a 50%-ethanol extract of the seeds. The DPPH and ABTS radical-scavenging activity and superoxide dismutase (SOD) activity were measured, and high antioxidant activity was seen in the ethyl acetate fraction. The antioxidant compounds in the ethyl acetate fraction were isolated using silica-gel column chromatography by adjusting the solvent between chloroform and methanol. Fraction numbers 2–7 showed activity of more than 50%. Next, primary column chromatography was used to mix and concentrate the fractions that demonstrated antioxidant activity. The fractions were then subjected to secondary column chromatography to obtain subfraction 4, which showed high antioxidant activity. The separation of subfraction 4 was then performed using high-performance liquid chromatography (HPLC). Three peaks were identified and peak number 2 was judged to be the primary antioxidative compound, which was then isolated by pure separation. Finally, the purified subfraction peak number 2 was identified as a fisetin compound by liquid chromatography–mass spectrometry (LC–MS), nuclear magnetic resonance (NMR), and HPLC.

## 1. Introduction

*Rhus verniciflua* (*R. verniciflua*), commonly known as lacquer tree, belongs to the family *Anacardiaceae*. It is native to China, Tibet (including the Himalayas), and the highlands of Central Asia. It is cultivated in Asian countries, including Korea, China, Japan, and Vietnam. There are about 600 species of the tree in the world and about 200 species are classified in the genus *R. verniciflua* [1,2]. *R. verniciflua* has been traditionally used as lacquered paint and as a medicinal plant in Korea, China, and Japan for thousands of years. In oriental medicine, it is known to be effective in eliminating eohyeol, promoting blood circulation, and treating mania, irregular menstruation, blood pressure, constipation, and abdominal pain [3,4]. It is also known to be used in a form of cooking chicken or duck [5]. The main components of *R. verniciflua* are urushiol, fustin, fisetin, sulfuretin, chalcone, and gallic acid. Urushiol is a catechol compound with two hydroxyl groups on the benzene ring, and it is composed of a long fatty-acid chain with 15 carbon atoms and a side-branch bonding structure. Urushiol derivatives vary, depending on the number of double bonds in the side branch and the type of bond [6,7]. The catechols in urushiol bind to and penetrate the skin, where they are oxidized to quinone intermediates and bind surface proteins on antigen-presenting cells in the epidermis, which in turn leads to an allergy cascade [8,9]. Several studies on the benefits of *R. verniciflua* on physiological processes have been reported. These include neuroprotective and anti-inflammatory effects [10], anti-platelet effects [11], antioxidant activity [12,13], and anti-cancer effects [14] in vitro, and anti-obesity effects [15] and anti-mutagenic effects [16] in vivo. Given the proven health benefits of *R. verniciflua*, studies have focused on the use of extracts from various parts of the plant as functional food materials. However, it was necessary to identify the plant parts with high urushiol content and find ways to reduce it. Urushiol is a primary component of xylem, and several studies have focused on reducing its content [17,18,19]. Furthermore, the seed of *R. verniciflua* is unique as a food material because of its low urushiol content and high antioxidant activity compared to other parts of the tree [20,21]. Therefore, more detailed studies on *R. verniciflua* seeds were needed. The goal of the study was to provide basic data for the development of functional food and health materials for *R. verniciflua* seeds. In this study, solvent fractionation was performed to isolate and identify antioxidants from the 50% ethanol extracts of *R. verniciflua* seeds, and then the antioxidant activity was measured. Open column chromatography separation was used to separate substances exhibiting antioxidant activity, and ^1^H and ^13^C-nuclear magnetic resonance (NMR) was performed to identify these substances.

## 2. Results and Discussion

### 2.1. Antioxidant Activity by Different Solvent Fractions

**Figure 1:**
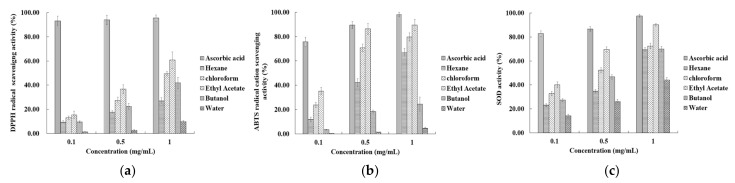
The antioxidant activities of the solvent fractions from *R. verniciflua*-seed extract with 50% ethanol. (**a**) DPPH free-radical-scavenging activity; (**b**) ABTS radical-cation-scavenging activity; (**c**) SOD activity. Data shown are means ± SD values of duplicate determinations from three separate experiments. DPPH: 2,2-diphenyl-1-picrylhydrazyl), ABTS: 2,2′-azino-bis-(3-ethylbenzothiazoline-6-sulfonic acid, SOD: superoxide dismutase, SD: standard deviation.

Figure 1a shows the results of the DPPH free-radical scavenging activity after performing hexane, chloroform, ethyl acetate, butanol, and water-solvent fractionation of the 50% ethanol extract of the *R. verniciflua* seed. At a concentration of 1 mg mL^−1^, the hexane, chloroform, ethyl acetate, butanol, and water contents were 26.87%, 49.55%, 60.73%, 41.79%, and 9.86%, respectively. The DPPH radical-scavenging activity of the ethyl acetate fraction was higher than those of the other fractions. The ABTS radical-scavenging activity results after performing hexane, chloroform, ethyl acetate, butanol, and water solvent fractionation of the 50% ethanol extract of *R. verniciflua* seed are shown in Figure 1b. At a concentration of 1 mg mL^−1^, the hexane, chloroform, ethyl acetate, butanol, and water contents were 66.87%, 79.79%, 89.48%, 24.38%, and 4.54%, respectively. The ABTS radical scavenging activity of the ethyl acetate fraction was higher than those of the other fractions. The SOD activity results after performing hexane, chloroform, ethyl acetate, butanol, and water solvent fractionation of the 50% ethanol extract of *R. verniciflua* seed are shown in Figure 1c. At a concentration of 1 mg mL^−1^, the hexane, chloroform, ethyl acetate, butanol and water contents were 69.19%, 72.50%, 90.15%, 69.93% and 44.19%, respectively. The antioxidant measurements, ABTS, DPPH radical-scavenging activity, and SOD activity, all showed a concentration-dependent increase. The ethyl-acetate fraction showed higher antioxidant activity than the other samples. Therefore, silica-gel column chromatography was performed using the ethyl-acetate fraction.

### 2.2. Antioxidant Activities of the Primary Fractions

**Figure 2:**
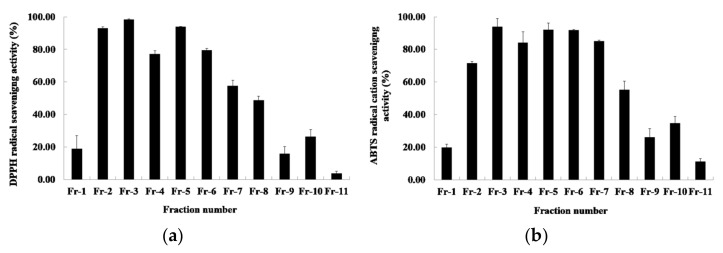
The antioxidant activities of *R. verniciflua*-seed extract by first silica-gel column chromatography. (**a**) DPPH free-radical-scavenging activity; (**b**) ABTS radical-cation-scavenging activity. Data shown are means ± SD values of duplicate determinations from three separate experiments. DPPH: 2,2-diphenyl-1-picrylhydrazyl), ABTS: 2,2′-azino-bis-(3-ethylbenzothiazoline-6-sulfonic acid, SD: standard deviation.

Among the fraction solvents, the ethyl acetate showed the highest antioxidant activity. The results of measuring the DPPH radical-scavenging activity of the ethyl acetate fraction by the first silica-gel column chromatography are shown in Figure 2a. The DPPH radical-scavenging activity of 11 fractions was between 3.76 and 93.93%. The fractions activity of showing 50% or more showed 93.11%, 98.38%, 77.26%, 93.93%, 79.61%, and 57.53%, respectively, from fraction numbers 2 to 7. The results of measuring the ABTS radical scavenging activity are shown in Figure 2b. The ABTS radical scavenging activity of the 11 fractions was between 11.39 and 94.06%. Among these, the fractions showing more than 50% activity were fraction numbers 2 to 8, which showed 71.51%, 94.06%, 84.12%, 92.06%, 91.75%, 85.06%, and 55.19%, respectively. The DPPH and ABTS radical scavenging activity measured by performing primary silica-gel column chromatography showed that fraction number 2 to 7 had high activity. Therefore, the fractions in this section were combined and subjected to secondary silica-gel column chromatography.

### 2.3. Antioxidant Activities of Secondary Fractions

**Figure 3:**
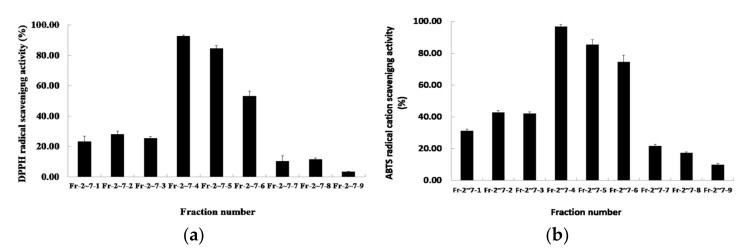
The antioxidant activities of *R. verniciflua*-seed extract by second silica-gel column chromatography. (**a**) DPPH free-radical-scavenging activity; (**b**) ABTS radical-cation-scavenging activity. Data shown are means ± SD values of duplicate determinations from three separate experiments. DPPH: 2,2-diphenyl-1-picrylhydrazyl), ABTS: 2,2′-azino-bis-(3-ethylbenzothiazoline-6-sulfonic acid, SD: standard deviation.

Primary column chromatography was used to mix and concentrate the fractions demonstrating antioxidant activity, after which secondary silica-gel column chromatography was carried out. A total of nine subfractions were obtained. The results of measuring the DPPH radical-scavenging activity of the secondary fraction are shown in Figure 3a. The DPPH radical-scavenging activity of the nine subfractions was between 3.35 and 92.75%, and subfraction number 4 showed the highest activity (92.75%). The ABTS radical-scavenging-activity results are shown in Figure 3b. The DPPH radical-scavenging activity of the nine subfractions was between 9.82 and 96.97%, and subfraction number 4 showed the highest activity (96.97%). Therefore, subfraction number 4 was used as the sample for HPLC separation.

### 2.4. Antioxidant Activities of HPLC Fractions

**Figure 4:**
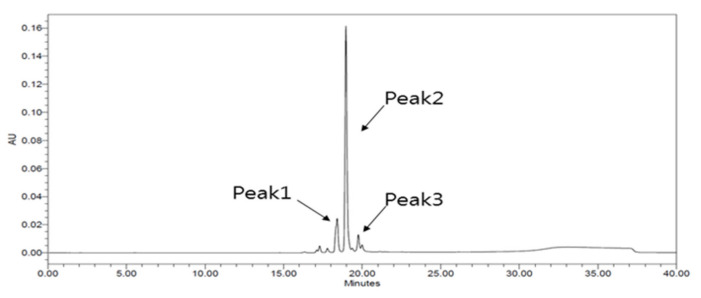
The HPLC chromatogram of subfraction number 4. HPLC: high-performance liquid chromatography.

A total of nine fractions were obtained by performing secondary silica gel column chromatography, and subfraction number 4 showed the highest antioxidant activity. Therefore, the HPLC separation of fraction number 4 was performed. As shown in Figure 4, a total of three peaks were identified; peak number 2 was identified as the primary compound, and it was isolated by pure separation.

### 2.5. Identification of Fraction Peak Number 2’s Substances by Liquid Chromatography–Mass Spectrometry (LC-MS), NMR, and HPLC

The structure of fraction peak number 2, separated by HPLC, was identified by LC-MS, NMR, and HPLC. A measurement using the LC_MS spectrum confirmed that the peak 2 compound was a pure peak. Its LRESIMS m/z data showed an [H] + 286.9 and [M + H] + 287.9 (Figure S1).

The ^1^H-NMR spectrum (400 MHz, DMSO-d_6_) showed d 10.73 (s, 1H), 9.49 (s, 1H), 9.26 (s, 1H), 9.03 (s, 1H), 7.90 (d, J = 9.6 Hz, 1H), 7.67 (d, J = 1.8 Hz, 1H), 7.52 (dd, J = 8.5, 2.1 Hz, 1H), and 6.91-6.85 (m, 3H)] (Figure S2), and the ^13^C-NMR spectrum (100, 125 MHz, DMSO-d_6_) was 102.19, 114.59, 115.03, 115.31, 115.94, 120.00, 122.86, 126.85, 137.56, 145.41, 147.62, 156.65, 162.63, and 172.34 (Figure S3). Its NMR data were identical to those of prior research [22,23]. Therefore, fraction peak number 2 was confirmed to be 3,3’,4’,7-tetrahydroxy flavones (fisetin) based on the peaks showing ^1^H-NMR and ^13^C-NMR spectra.

**Figure 5:**
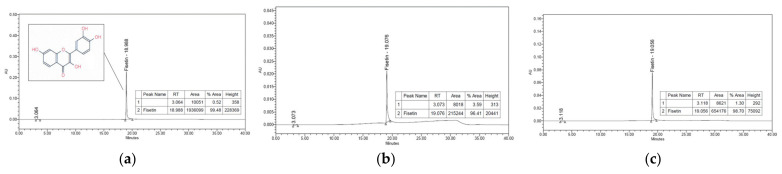
Fraction compound of *R. verniciflua*-seed ethanol extract identified by high-performance liquid chromatography. (**a**) Fisetin (Standard, Sigma-Aldrich Inc., St. Louis, MO, USA); (**b**) sample (fraction of *R. verniciflua* seed 50% ethanol extract); (**c**) spike test of sample and fisetin.

Finally, a HPLC analysis was performed to confirm the identity of the antioxidant compound fraction number 2 corresponding to the structure identified by the LC–MS and NMR measurements, as shown in Figure 5.

The antioxidant substance isolated from the *R. verniciflua* seed was confirmed to be fisetin through LC–MS and NMR identification, after which the samples and standards were analyzed by HPLC to confirm that they were fisetin compounds.

Fisetin (3,3’,4’,7-tetrahydroxy flavones) is a commonly occurring flavonoid found in various plants, such as apples, strawberries, and *R. verniciflua* [24,25]. Fisetin has also been reported to possess various biological properties, with significant health benefits [26], such as antioxidant activity [27,28], anticancer effects [29,30], antitumor activity [31], and the inhibition of adipogenesis [32].

## 3. Materials and Methods

### 3.1. Materials and Chemicals

The fruit of the *R. verniciflua* variety used in this experiment was collected from Imsil-gun (35°40′07” N; 127°12′28” E and altitude 243 m), Jeollabuk-do, South Korea. The seeds were used after removing the outer and the middle skin of the fruit. All the solvents used for the extraction and fractionation procedures (hexane, chloroform, ethyl acetate, butanol, and water) were purchased from J.T. Baker (Radnor, PA, USA). The 1,1-Diphenyl-2-picrylhydrazyl (DPPH), 2,2-azino-bis-(3-ethylbenzothiazoline-6-sulfonic acid) diammonium salt (ABTS), and other chemical reagents were analytical grade. Superoxide dismutase (SOD) activity assay kits were purchased from BioVision (BioVision Inc., Milpitas, CA, USA). All reagents for the analyses were purchased from Sigma-Aldrich (St. Louis, MO, USA).

### 3.2. Preparation of R. Verniciflua Seed Extracts Using 50% Ethanol as a Solvent

For extraction, 10 L of 50% ethanol was added to 1 kg of *R. verniciflua*-seed samples and triturated with a homogenizer, followed by extraction by stirring and leaching at room temperature for 24 h, and then filtered with Whatman filter paper grade no. 2. The extraction filtrate was concentrated under reduced pressure using a rotary vacuum evaporator (EYELA, NE-1001, Tokyo, Japan) at 37 °C in a water bath, and freeze-dried.

### 3.3. DPPH Radical-Scavenging-Activity Assays

The DPPH radical-scavenging activity was measured by modifying Blois’ method [33]. Briefly, the samples to be tested (100 μL) were added to 150 μL of ethanolic DPPH (0.3 mM). The reaction mixtures were shaken vigorously and maintained at indoor temperature for 30 min while avoiding light. The absorbance was determined at 530 nm using a spectrophotometer (Biochrom, Libra s22, Cambridge, UK). All determinations were performed in triplicate.

### 3.4. ABTS Radical-Scavenging-Activity Assays

The ABTS radical-scavenging activity was measured according to the method of Re et al. [34]. The ABTS radical cation was prepared by mixing equal volumes of 7 mM ABTS solution and 2.45 mM potassium persulfate. The mixture was incubated for 24 h at room temperature in the dark to yield a dark-colored solution containing ABTS radicals and then diluted with distilled water to an absorbance of 0.7 ± 0.02 units at 630 nm. Extracts (100 μL) were allowed to react with 100 μL of ABTS solution for 30 min in the dark until a stable absorbance was obtained. The reduction in absorbance at 630 nm was measured using a spectrophotometer (Biochrom, Libra s22, Cambridge, UK). Data for each assay were recorded in triplicate.

### 3.5. Superoxide Dismutase (SOD) Assays

Superoxide dismutase (SOD) catalyzes the dismutation of the superoxide anion into hydrogen peroxide and molecular oxygen. The rate of the reduction with a superoxide anion is linearly related to the xanthine oxidase activity and is inhibited by SOD [35]. The SOD activity was measured by adding the samples to be tested (20 μL) to 20 μL of H_2_O, water-soluble tetrazolium salt (WST) working solution 200 µL, SOD dilution buffer 20 µL, and SOD enzyme solution 20 µL each. The reaction mixtures were allowed to react at 37 °C for 20 min. Absorbance was determined at 450 nm using a spectrophotometer (Biochrom, Libra s22, Cambridge, UK). All determinations were performed in triplicate.

### 3.6. Separation by Different Solvent Fractions

To obtain solvent fractions, distilled water was added to the 50% ethanol extracted dried product, after which hexane solvents were added to the separating funnel at a 1:1 ratio and the hexane layer was separated using a fractionated funnel shaker. In the same process, chloroform, ethyl acetate, n-butanol, and water layers were sequentially added to obtain each fraction.

### 3.7. Separation by Silica-Gel Column Chromatography

Primary silica-gel column chromatography was carried out using silica gel (70–270 mesh, Merck, Darmstadt, Germany) and a glass column (∅25 × 300 mm) to isolate the active substances in the ethyl acetate fraction. The ethyl acetate fraction was injected into a glass column containing silica gel, and 23 fractions were obtained by sequentially adjusting the solvent from chloroform: methanol (100:1) to chloroform: methanol (1:100). The spectrum measured in the wave scan mode using a spectrophotometer (Biochrom, Libra s22, Cambridge, UK) and the fractions separated by thin-layer chromatography (TLC) were compared. The fractions were further divided into 11 fractions and then concentrated under reduced pressure. For the 11 fractions, the DPPH and ABTS radical-scavenging activities were measured, and fractions showing 50% and 60% or more of antioxidant activity, respectively, were collected, concentrated under reduced pressure, and then subjected to secondary column chromatography (sequentially adjusting the solvent from chloroform:methanol (100:1) to chloroform:methanol (60:40)).

### 3.8. Separation by High-Performance Liquid Chromatography (HPLC)

Separation of the antioxidant active components by HPLC (Waters 600E system, Waters Co., Milford, MA, USA) was performed as follows: The fractions demonstrating antioxidant activity according to secondary column chromatography were concentrated under reduced pressure, after which antioxidant components were isolated from the concentrated solution using HPLC and μ-Bondapak C_18_ column chromatography (ID 30 × 300 mm, 15–20 μm, Waters Co., Milford, MA, USA). The mobile phase was a gradient-elution system consisting of 0.1% formic acid (A) and methanol (B), with a linear gradient from 0 to 100% B, a flow rate of 15 mL min^−1^, and injection volume of 0.5 mL.

### 3.9. Identification of Fraction Peak 2 Substance by HPLC–Mass Spectrometry (MS)

The HPLC–MS analysis of separated peak 2 was performed using a Poroshell 120 column (EC-C_18_, 2.7 μm 50 × 3.0 mm, Agilent Technologies, Santa Clara, CA, USA) on an Agilent Technologies 1260 series, 6130 Quadrupole LC-MS, and 1260 Hip ALS. The column temperature was set at 37 °C. The gradient-elution system consisted of water (A) and acetonitrile (B), with a linear gradient from 0 to 20% B, flow rate of 0.8 mL min^−1^, and injection volume of 30 µL.

### 3.10. Identification of Antioxidant Active Substances by Nuclear Magnetic Resonance (NMR)

The structure of the material was confirmed using NMR spectra, recorded on a JNM-ECZS series FT 400 NMR (JEOL, Akishima, Japan) spectrometer and Varian Inova spectrometers using dimethysulfoxide-d6 as the solvent, obtained from Cambridge Isotope Laboratories (CIL), Inc. Chemical shifts were reported with reference to the respective solvent peaks.

### 3.11. Identification of Active Antioxidant Substances by HPLC

To confirm the identity of the antioxidant compounds corresponding to the structure identified by the NMR measurement, HPLC was performed using Lee et al.’s method [36]. Samples and standards were added to the methanol, followed by filtration through a 0.45-μm membrane filter (Millipore Co., Burlington, MA, USA) and analysis by HPLC (Waters e2695, Waters Co., Milford, MA, USA). The column comprised Capcell pak C_18_ UG120, S5 (4.6 × 250 mm) (Shiseido co., LTD, Tokyo, Japan). The eluents were 0.1% formic acid in water (A) and 0.1% formic acid in 90% acetonitrile with water (B) and the flow rate used was 0.1 mL/min. The gradient was as follows: 0–4 min, 10% B; 4–20 min, 60% B; 20–23 min, 10% B; 23–30 min, 10% B. The injection volume was 10 µL and the detection wavelength was 310 nm (Waters 2489 UV/visible detector, Waters Co., Milford, MA, USA).

## 4. Conclusions

This study confirmed that *R. verniciflua* seeds contain fisetin. Hence, the results of this study provide basic data for the development of functional food and health materials for *R. verniciflua* seeds and contribute to the development of various products.

## Data Availability

The authors confirm that the data supporting the findings of this study are available within the article.

## Supplementary Materials

The following supporting information can be downloaded at: https://www.mdpi.com/article/10.3390/molecules27144510/s1. Figure S1: LC-MS spectrum of fisetin. Figure S2: ^1^H NMR spectrum of fisetin. Figure S3: 13C NMR spectrum of fisetin.
