# Supplementary material for: Isolation and Identification of Fisetin: An Antioxidative Compound Obtained from Rhus verniciflua Seeds
Source: Molecules. 2022 Jul 14;27(14):4510. doi: 10.3390/molecules27144510 (PMC9318972; doi:10.3390/molecules27144510)
Supplement: Supplementary file 1 [file molecules-27-04510-s001.zip › molecules-1786810-supplementary.pdf]

molecules

Isolation and Identification of Fisetin an Antioxidative Compound Obtained from *Rhus verniciflua* Seeds

Su Hwan Kim [1] and Chang Ki Huh [2,*]

[1] Research Institute of Food Industry, Sunchon National University, Suncheon 57922, Korea; Suhwan010@sunchon.ac.kr
[2] Department of Food Science and Technology, Sunchon National University, Suncheon 57922, Korea; hck1008@sunchon.ac.kr
* Correspondence: hck1008@sunchon.ac.kr; Tel.: +82-61-750-3251

Contents

Figure S1. LC-MS spectrum of fisetin

Figure S2. ^{1}H NMR spectrum of fisetin

Figure S3. ^{13}C NMR spectrum of fisetin

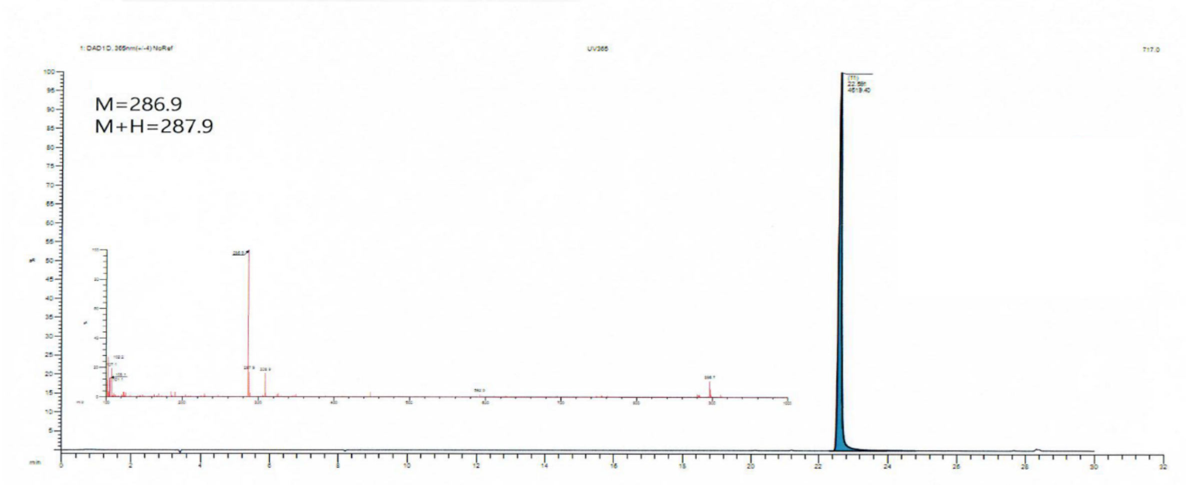

Figure S1. LC-MS spectrum of fisetin.

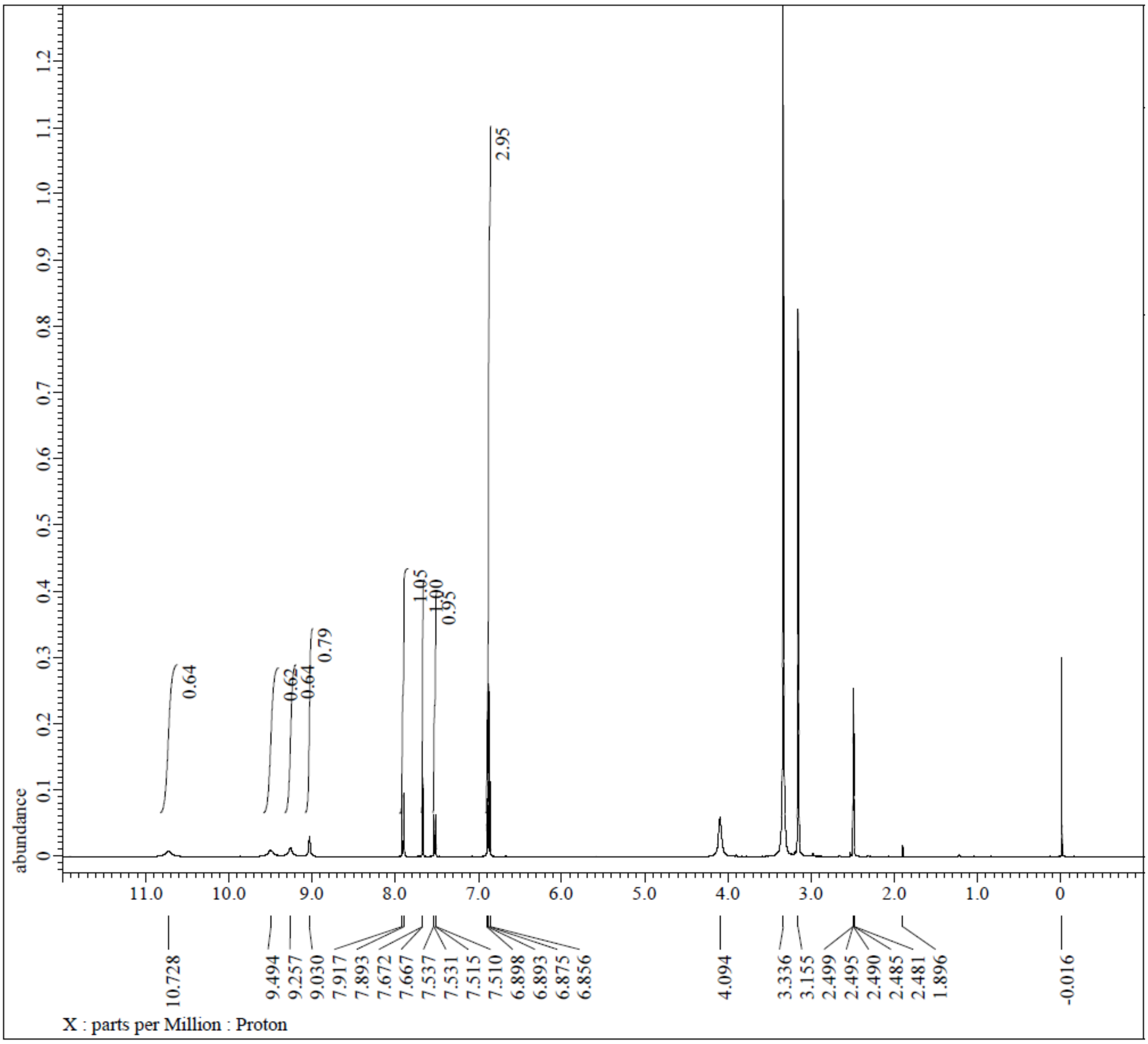

Figure S2. ^{1}H NMR spectrum of fisetin.

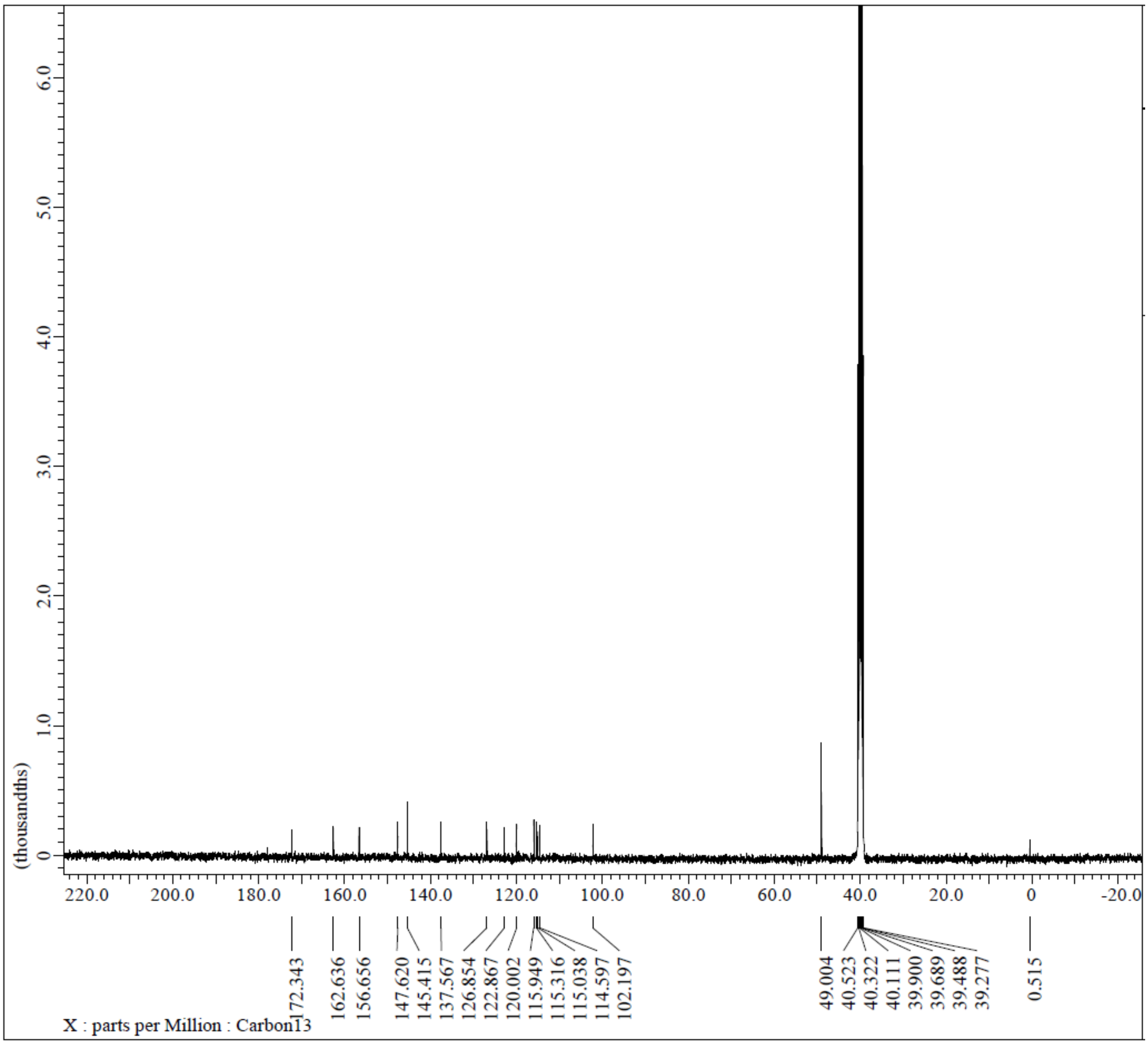

Figure S3. ^{13}C NMR spectrum of fisetin.
